# Case Report: Primary Indolent Epstein-Barr Virus-Positive T-Cell Lymphoproliferative Disease Involving the Central Nervous System

**DOI:** 10.3389/fsurg.2022.775185

**Published:** 2022-03-02

**Authors:** Kun Wang, Jinjian Li, Xuehui Zhou, Junhui Lv, Yirong Wang, Xinwei Li

**Affiliations:** ^1^Department of Neurosurgery, Hangzhou Xiasha Hospital, Sir Run Run Shaw Hospital, Medical College, Zhejiang University, Hangzhou, China; ^2^Department of Neurosurgery, Sir Run Run Shaw Hospital, Zhejiang University School of Medicine, Hangzhou, China; ^3^Department of Emergency, Shangrao Hospital of Nanchang University, Jiangxi, China; ^4^Department of Neurosurgery, Sir Run Run Shaw Hospital, Medical College, Zhejiang University, Hangzhou, China

**Keywords:** EBV, central nervous system (CNS), T-cell lymphoproliferative disease, adult, LPD

## Abstract

**Background:**

T-cell lymphoproliferative disease (T-LPD), characterized by primary Epstein–Barr virus (EBV) infection and clonal proliferation of T cells, occurs both in systemic and non-lymphatic organs. However, isolated indolent EBV-positive T-LPD involving the central nervous system has not been reported.

**Case Presentation:**

A 48-year-old male who complained of headache, blurred vision, and weakness of the left lower limb for 1 month was hospitalized in our department. Neither neurological deficit nor palpable lymphadenopathy had been found. Bone marrow and laboratory tests had shown no abnormality as well. Enhanced MRI demonstrated enhanced cotton-like lesions up to 20 mm in diameter located in the right frontal, temporal, parietal and left parietal, occipital lobes with perifocal edema. Neuronavigation-assisted mini-craniotomy was performed to achieve total excision of the right temporal superficial lesion and identify the diagnosis. Pathological and EBV analysis described the lesion as indolent EBV-positive T-cell lymphoproliferative disease of the central nervous system (CNS). Then, a therapeutic regimen including whole-brain irradiation, chemotherapy, prednisolone, and aciclovir was given. Serial radiological imaging showed no signal of recurrence at 5 months' follow-up.

**Conclusion:**

Primary indolent T-LPD in the central nervous system is quite rare, and it needs to be distinguished from aggressive cerebral T-cell lymphoma, metastatic tumors, and other CNS lesions.

## Introduction

T-cell lymphoproliferative disease (T-LPD) is an exceptionally rare disorder characterized by primary EBV infection and clonal proliferation of T cells. It is more frequent in children or young people than in adults and has an aggressive clinical course (1, 2). Cases of it occurs in both systemic (2) and non-lymphatic organs such as the gastrointestinal tract (3, 4), and most of them are secondary to immune deficiency diseases or organ transplants (1, 5). However, primary cerebral T-LPD has not been reported. Herein, we present an uncommon case of indolent EBV-positive T-LPD, which originally occurred in the central nervous system.

## Case Presentation

**Figure 1:**
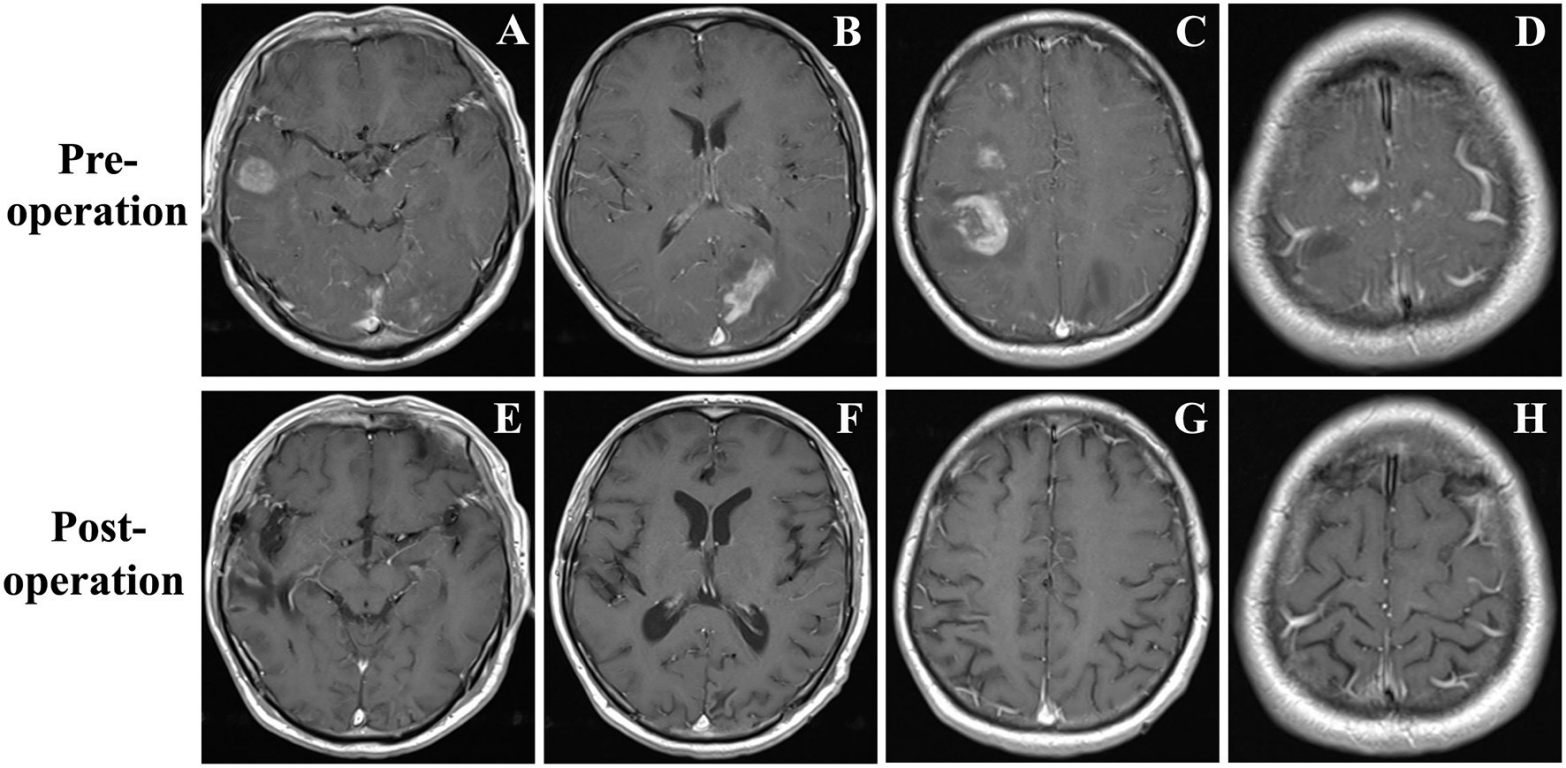
**(A–D)** Brain MRI demonstrated a few contrast-enhancing, cotton-like lesions up to 25 mm in diameter located in the right temporal, frontal, and parietal lobes, and left occipital lobe with perifocal edema. **(E–H)** Brain MRI revealed all the lesions disappeared at the fifth month of follow-up.

A 48-year-old male was admitted with headache, blurred vision, and left lower limb weakness. Neither neurological deficit nor palpable lymphadenopathy had been found before. Enhanced MRI revealed cotton-like lesions up to 20 mm in diameter that were located in the right frontal, temporal, parietal and left parietal, occipital lobes with perifocal edema (Figures 1A–D). Bone marrow and laboratory tests had also shown no abnormality.

**Figure 2:**
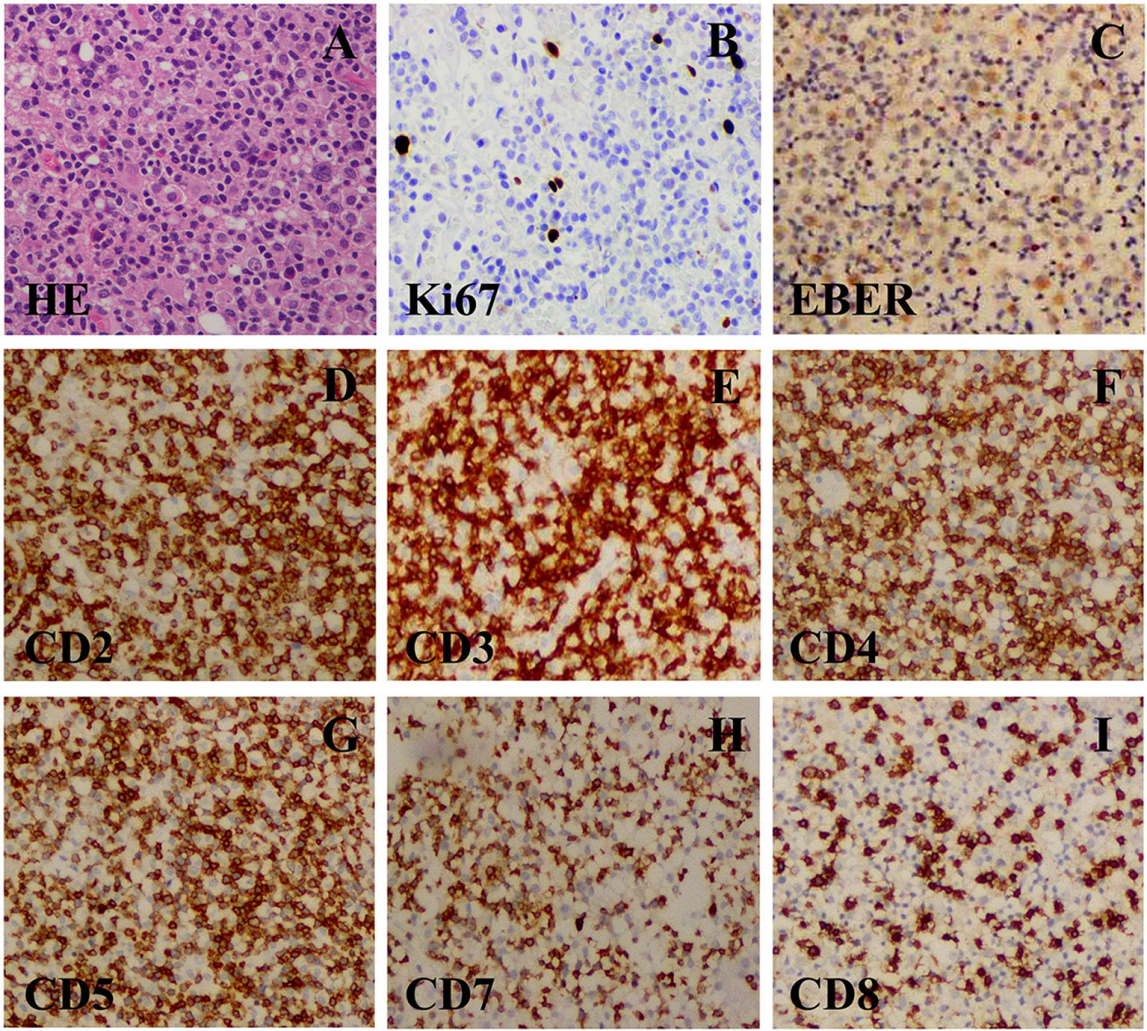
**(A)** A resection specimen showed a lot of densely small lymphoid cells diffuse infiltrating. The small lymphoid cells were predominantly composed of polymorphous cells with hyperchromatic nuclei with sporadic mitoses (original magnification ×400). **(B–G)** Immunohistochemical staining (original magnification ×400). **(B)** CD3(+) T-cell. **(C)** CD5(+) B-cell. **(D)** CD7(+) B-cell. **(E)** CD8(+) B-cell. **(F)** CD20(–) B-cell. **(G)** CD56(–) NK-cell. **(H)** Ki67(+). **(I)** Ki67(+) (about 40%).

Based on these clinical data, diagnoses of central nervous system lymphoma and metastatic tumors were first suspected. Neuronavigation-assisted mini-craniotomy was decided to performed to achieve total excision of the right temporal superficial lesion and to confirm the diagnosis. Histological analysis revealed a dense infiltrate of small lymphoid cells, consisting of polymorphous cells with hyperchromatic nuclei (Figure 2A). Immunohistochemistry revealed that the infiltrated cells were positive for CD2, CD3, CD4, CD5, CD7, and CD8 (Figures 2B–D,F–H), and negative for CD20, CD23, CD30, CD56, T-cell-restricted intracellular antigen 1 (TIA-1), cyclin D1, BCL-2, GFAP, and granzyme B. The Ki-67 labeling index was low (<5%) (Figure 2E). EBV-encoded RNA (EBER) *in situ* hybridization was performed in this case and showed evidence of EBV infection. EBV was further detected in the serum (EBV-CA-IgG > 750 U/ml, EB-DNA: 1.09 × 104 copies/ml). The pathological diagnosis of indolent EBV-positive T-cell lymphoproliferative disease in the central nervous system was made ultimately.

Then, fractionated low-dose whole-brain irradiation (6MV-X SAD 100 DT 3000cGy/15f/22d) was given. Chemotherapy was applied after completion of radiotherapy (bortezomib 2 mg d1, d4, d8, d11 + methylprednisolone 50 mg Q12 d1–d14). The patient was asked to stop chemotherapy because of diarrhea during the third chemotherapy (the 4th day of chemotherapy) session. The patient was followed regularly by an outpatient review, and there were no obvious signs of recurrence at the fifth month of follow-up (Figures 1E–H).

## Discussion

Indolent EBV-positive T-LPD is a new subgroup in the 2016 revision of the World Health Organization Classification (6) that presents a great challenge to society due to high disability. Indolent EBV-positive T-LPD is considerably more prevalent in Eastern Asian countries (7). The most commonly involved sites of EBV-associated LPD are the lymph nodes, skin, liver, spleen, bone marrow (2), upper aero digestive tract (e.g., the nasal cavity and nasopharynx), and paranasal sinuses (3).

Clinically, the course of primary T-cell LPD in the CNS is still unclear, and there have been no relevant reports. EBV infection may play an important role in the progression from benign to malignant proliferation (2, 7–9). EBV is known to be associated with infectious mononucleosis (IM) and several lymphoid malignancies (10). In addition, EBV infection is strongly associated with stage of development between tumor and non-cancer in certain LPDs (11). Ohshima et al. described that EBV+-T-LPD is essentially a disease spectrum that incorporates different stages of development (7). Miyuki et al. (2) made a summary of 16 reported cases with systemic EBV+ T-cell LPD. However, Mineui Hong suggested that indolent T-cell LPD in the GI tract is a non-progressive clonal disorder and has a persistent lesion for several years without progression (12). The different results may be related to different sites and progression stages of T-LPD, which made the diagnosis of the disease more difficult. Therefore, physicians rely on pathological examination and immunohistochemical staining for the correct diagnosis.

Although cerebral T-cell lymphomas are frequently reported (8, 13), there are no reports on T-LPD in the CNS. This case was clinically characterized by EBV+ T-cell proliferation in the CNS, with an indolent period and developed a progressive course soon.

This is a rare case of primary cerebral indolent T-cell proliferative disease that needs to be distinguished from T-cell lymphoma. Because of the similarity in clinical and radiographic findings, T-LPD can be easily misdiagnosed as T-cell lymphoma. Histopathology remains the gold standard in diagnosis (8). Although the histological analysis in this case revealed abnormal T-cell proliferation, the infiltrated cells in our case had strong, diffuse positivity for CD2, CD3, CD4, CD5, CD7, and CD8, while CD56, CD30, T cell receptor (TCR), and TIA-1 were negative, which did not reach the diagnostic criteria of T-cell lymphoma according to National Comprehensive Cancer Network (NCCN) Guidelines Version 1.2021 T-Cell Lymphomas. The case showed a high EBV-DNA load, and EBER was positive; the cells were confirmed as T- cells. Therefore, we referred to this patient as having EBV-positive T-LPD rather than T-cell lymphoma.

Although the low expression of Ki-67 means poor proliferative activity of tumor cells, the clinical behavior was not silent because of increased intracranial pressure and neurological deficits. The lesions responded transiently to radiotherapy after being diagnosed but relapsed shortly. Chemotherapy including bortezomib and methylprednisolone is an effective therapy to these elapsed lesions, but long-term effects were not observed because of serious complications. Consequently, a strategy including corticosteroids, chemotherapy, and irradiation was effective to primary cerebral T-cell LPD.

In addition, delay is diagnosis is inevitable in this case. Firstly, we discern that it was craniotomy rather than stereoscopic biopsy for the consideration of accessibility of superficial lesions and the influence of pre-treatment's corticosteroids (5 days during pre-operation), which may be led to misdiagnosis or delayed diagnosis. The second reason was minimal cytological atypia of the proliferative lymphocytes and a series of immunohistochemical staining. We lack the experience and self-confidence because of its anomaly morbidity on the one hand; on the other hand, the pathological result of rapid freezing during the operation was not clear. After the first craniotomy, it took more than one month to summarize the conclusions from different medical centers before making the diagnosis. Furthermore, 3 weeks was spent to confirm the initial diagnosis when it relapsed. A multidisciplinary discussion or consultations can help to get an accurate early diagnosis.

## Conclusion

So far, as we know, this is the first case of primary indolent T-cell LPD in the CNS published in literature, and it needs to be distinguished from aggressive cerebral T-cell lymphoma. A strategy including corticosteroids, chemotherapy, and irradiation was effective, but long-term effects remained unclear.

## Data Availability Statement

The original contributions presented in the study are included in the article/supplementary material, further inquiries can be directed to the corresponding author.

## Ethics Statement

This study was approved by the Ethics Committee of Sir Run Run Shaw Hospital, Medical College, Zhejiang University (Hangzhou, China). The family of the patient signed the informed consent containing a detailed description of the purpose of this study. All the specimens were handled and made anonymous to meet the ethical and legal standards.

## Author Contributions

KW, XL, and JLi: writing—original draft. XL and JLi: review and editing. KW, JLi, XL, and JLv: project administration. YW and KW: supervision. XZ: review and editing and project administration and correction. All authors contributed to the article and approved the submitted version.

## Funding

This study was supported by the Natural Science Foundation of Zhejiang Province of China (Nos. LY20H160034, LY14H160025, and LY14H160017), Program for Health and Family Planning Commission of Hangzhou Municipality (No. A20210525), and National Natural Science Foundation of China (No. 81402044).

## Conflict of Interest

The authors declare that the research was conducted in the absence of any commercial or financial relationships that could be construed as a potential conflict of interest.

## Publisher's Note

All claims expressed in this article are solely those of the authors and do not necessarily represent those of their affiliated organizations, or those of the publisher, the editors and the reviewers. Any product that may be evaluated in this article, or claim that may be made by its manufacturer, is not guaranteed or endorsed by the publisher.
